# Mobile Apps for Increasing Treatment Adherence: Systematic Review

**DOI:** 10.2196/12505

**Published:** 2019-06-18

**Authors:** Virtudes Pérez-Jover, Marina Sala-González, Mercedes Guilabert, José Joaquín Mira

**Affiliations:** 1 Departamento Psicología de la Salud Universidad Miguel Hernández Elche Spain

**Keywords:** mobile health, medication alert systems, medication adherence

## Abstract

**Background:**

It is estimated that 20% to 50% of patients do not take their medication correctly, and this leads to increased morbidity and inefficacy of therapeutic approaches. Fostering treatment adherence is a priority objective for all health systems. The growth of mobile apps to facilitate therapeutic adherence has significantly increased in recent years. However, the effectiveness of the apps for this purpose has not been evaluated.

**Objective:**

This study aimed to analyze whether mobile apps are perceived as useful for managing medication at home and if they actually contribute to increasing treatment adherence in patients.

**Methods:**

We carried out a systematic review of research published using Scopus, Cochrane Library, ProQuest, and MEDLINE databases and analyzed the information about their contribution to increasing therapeutic adherence and the perceived usefulness of mobile apps. This review examined studies published between 2000 and 2017.

**Results:**

Overall, 11 studies fulfilled the inclusion criteria. The sample sizes of these studies varied between 16 and 99 participants. In addition, 7 studies confirmed that the mobile app increased treatment adherence. In 5 of them, the before and after adherence measures suggested significant statistical improvements, when comparing self-reported adherence and missed dose with a percentage increase ranging between 7% and 40%. The users found mobile apps easy to use and useful for managing their medication. The patients were mostly satisfied with their use, with an average score of 8.1 out of 10.

**Conclusions:**

The use of mobile apps helps increase treatment adherence, and they are an appropriate method for managing medication at home.

## Introduction

### Background

The World Health Organization has classified the lack of treatment adherence as a major global problem [1]. This is partly because of therapeutic nonadherence being associated with high health costs because of rehospitalizations as a consequence of the lack of therapeutic response, changes in prescriptions for other more potent and toxic medications that increase the risk of producing side effects or long-term medication dependence, and, above all, the decreased efficacy from medication that patients either do not take or take inappropriately [2,3]. These consequences lead to increased morbidity and mortality in nonadherent patients [2,4].

### Figures for Therapeutic Nonadherence

It is estimated that 20% to 50% of patients do not take their medication correctly [5-7]. The reasons for this lack of adherence to treatments are varied. On the one hand, patients may voluntarily stop taking their medication because of, for example, a perception of the lack of improvements, beliefs that they have not been diagnosed correctly, or the adverse effects of the drug. However, the most frequent reasons for therapeutic nonadherence are involuntary causes, such as confusion or simple forgetfulness [2,5,8,9]. Medication errors at home are more usual than expected by health care professionals [8,10,11]. This would seem to indicate that designing and applying methods that foster treatment adherence and contribute to reduce medication errors at home are necessary.

### Pillbox

The most commonly used device to promote medication adherence is the pillbox. People can independently manage their medications, check whether they have taken them or not, avoid the risk of taking them twice or not taking them at all, and reduce the rate of medication errors. Previous studies found that people who used a pillbox had better treatment adherence [12-14]. There are Medication Event Monitoring Systems (MEMSs), whose popular name is electronic pillbox. They have the additional feature of reminding patients to take the medication with alarms, and they are considered as the gold standard for measuring adherence [15]. However, unfortunately, none of these pillboxes are exempt from problems. They are too big to get them out of the house. In addition, patients have to understand the prescribed therapeutic regimen to organize the medication in the compartments and know how to manage these pillboxes [12-17].

### Smartphones and Health Apps

All the research indicates that new information technologies have been rapidly accepted by the entire population [18]. In the case of Spain, 94.6% of its population currently uses a mobile phone [19].

This boom in mobile phones has resulted in these devices being used to devise new procedures to promote therapeutic adherence. At first, short message service (SMS) text messages were sent and telephone calls were made to remind users of the need to take medication. These kinds of reminders have been very effective methods and are well accepted by patients [20-22].

Then, with the advent of smartphones came mobile apps that have also afforded new opportunities for carrying out actions that simplify daily tasks, among them caring for health [6,8,18,23-25]. Currently, there are more than 165,000 apps designed for these devices that are related to health, and one in 5 people have downloaded a mobile health (mHealth) app [18]. Among these apps are a growing number intended to help patients in the management of their disease and their medication, remind users to take their drugs, and provide them with information about how they should do it to promote treatment adherence. These mobile apps are not only intended to help people remember to take the medication, such as the electronic pillbox; they have additional useful features that not only promote medication adherence but also increase treatment adherence.

However, very little research has been undertaken to evaluate the effectiveness of these apps for the purposes for which they were intended or the level of acceptance among users [6,18,26]. There are also no studies about their contribution to safe medication use.

### Objective of This Study

This study aimed to analyze whether mobile apps that help people manage their medication in the home contribute to increasing patient adherence and are considered useful by the users.

## Methods

A systematic review study that applied the recommendations in the Preferred Reporting Items for Systematic reviews and Meta-Analysis declaration for these types of studies was carried out [27].

### Concepts to Be Taken Into Account in This Review

#### Pillbox

A small container that pills are carried in. A pillbox can make the medication task easier because it helps people to manage their daily medication. This device is associated with improvements in medication adherence and, subsequently, with better health [12].

#### Electronic Pillbox

The MEMS is a pill organizer that has the additional feature of reminding you to take your drugs with visual and audio alerts. This MEMS provides information about treatment adherence. Therefore, it is the gold standard for this purpose [17].

#### Mobile Apps

Mobile apps are computer programs or software installed on mobile electronic devices that supports a wide range of functions and uses, including television, telephone, video, music, word processing, and internet services [16].

#### Mobile Apps to Improve Medication Adherence

In this study, we considered the kind of mobile apps that help people to manage their medication. These mobile apps, compared with pillboxes or electronic pillboxes, have the main advantage of being a system that is incorporated into our smartphones [16].

### Selection of Studies

The inclusion criteria for this review included research published in either English or Spanish that provided results about the effectiveness or treatment adherence in using mobile apps in the management of medication in the home, with any age group as the study population and regardless of the pathology and prescribed medication. Both quantitative and qualitative research were included, as well as research with descriptive and experimental approaches. The studies included in this review included presentations of results about the effectiveness in fostering adherence to treatment, safe medication use, viability, acceptance, satisfaction, and usefulness of these mobile apps. We excluded studies that were merely descriptive about the design of the mobile apps without presenting the results of use experience. We also excluded studies in which the interventions to remind patients to take their medication were delivered via SMS text messaging, phone calls, or electronic pillboxes.

### Search Strategy

We carried out a search for scientific documentation in the Scopus, Cochrane Library, ProQuest, and MEDLINE databases using keywords associated with pillbox and mobile apps and using the Boolean indicators OR and AND (*pillbox* OR *pill reminder* OR *pill organizer* OR *pill dispenser* OR *medication organizer* OR *medication reminder* OR *medication systems* OR *medicine reminder* OR *reminder system* AND *mhealth* OR *mobile app* OR *mobile application*). The search for documents was limited to publications that appeared in scientific journals from January 2000 through January 2017. The same descriptors were used to search the internet for relevant gray literature using the Google search engine. We similarly undertook a manual search using the bibliographic references of the selected publications.

**Figure 1:**
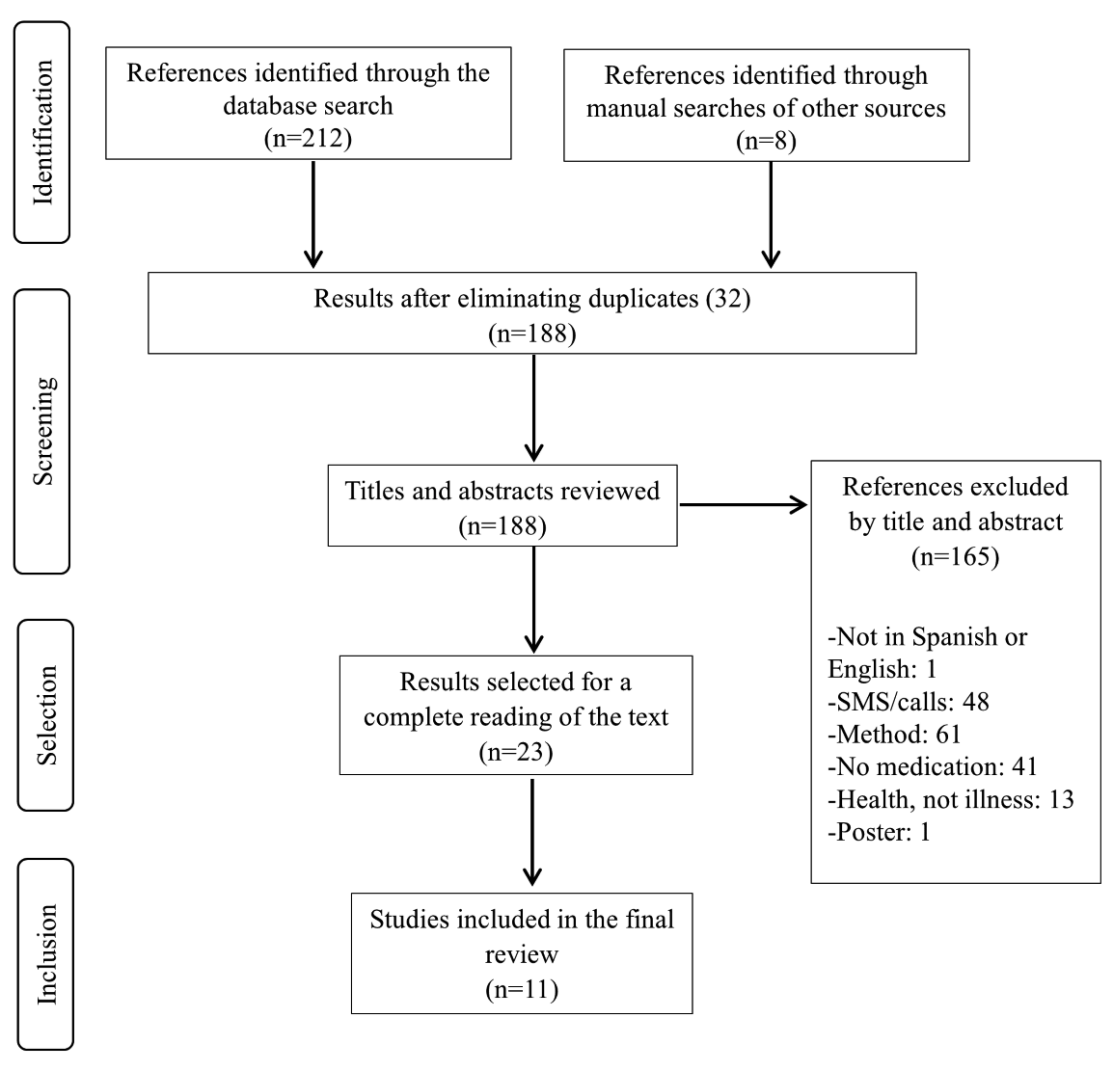
Flow diagram of the study inclusion and exclusion process.

The initial search identified 212 papers, of which 32 were eliminated because of being duplicates. Similarly, we found an additional 8 studies within either the bibliographies of the articles selected or through a Google search. We analyzed the titles and abstracts and eliminated 188 papers because they did not fulfill the inclusion criteria. We then fully read the 23 remaining papers and discarded 10 of them because they did not evaluate the effectiveness of the mobile app. Ultimately, 11 papers fulfilled the inclusion criteria (Figure 1).

### Data Extraction

The data extracted for each study included its country, objective, participants, chronic condition, design, and duration. Moreover, we recorded the functions of each mobile app, names of its designer(s), measures for evaluating adherence, measures for evaluating the mobile app, and outcomes of its evaluation.

In addition, to evaluate the quality of the reviewed publications, we first analyzed the level and degree of evidence following the classification proposed by the Scottish Intercollegiate Guidelines Network [28]. Then, we assessed the following criteria (with a dichotomous yes/no scale): if it was reflected in the study that patients had participated to some degree in the app design, if the sample error had been controlled by adjusting the size of the sample under study, if there had been randomization with the samples who participated in the study to determine the app effectiveness, if validated measuring scales had been used, and whether the app had been used under natural conditions for periods of time exceeding 3 months.

We classified the levels of evidence as follows: 1++ (meta-analyses, systematic reviews of clinical trials, or high-quality clinical trials with very little risk of bias), 1+ (meta-analyses, systematic reviews of clinical trials, or well-conducted clinical trials with little risk of bias), 1− (meta-analyses, systematic reviews of clinical trials, or clinical trials with high risk of bias), 2++ (systematic reviews of cohort or case-control studies or studies of high-quality diagnostic tests and cohort or case-control studies of high-quality diagnostic tests with very little risk of bias and a high probability of establishing a causal relationship), 2+ (cohort or case-control studies or studies of well-conducted diagnostic tests with a low risk of bias and a moderate probability of establishing a causal relationship), 2− (cohort or case-control studies with a high risk of bias), 3 (nonanalytical studies, such as case reports and case series), and 4 (expert opinions) [28].

We classified the strengths of the recommendations as (A) at least 1 meta-analysis or systematic review of a controlled and randomized trial (CRT) or a level 1++ CRT, directly applicable to the target population or sufficient evidence extrapolated from 1+ level studies, directly applicable to the target population and whose results demonstrate overall consistency; (B) sufficient evidence deriving from level 2++ studies, directly applicable to the target population and whose results demonstrate overall consistency, with evidence extrapolated from either 1++ or 1+ level studies; (C) sufficient evidence deriving from level 2+ studies, directly applicable to the target population and whose results demonstrate overall consistency, with evidence extrapolated from level 2++ studies; and (D) evidence from either level 3 or 4 studies, with evidence extrapolated from level 2+ studies [28].

The evaluation and classification of the studies found during the search strategy were completed independently by 2 investigators (MS and VPJ). Discordant elements were discussed by both investigators until an agreement was reached.

## Results

The initial search identified 212 papers. Ultimately, 11 papers fulfilled the inclusion criteria (Figure 1). Multimedia Appendix 1 shows the level of evidence and the degrees of recommendation of each of the 11 selected studies. Multimedia Appendix 2 shows the assessment of the internal quality of the design of each study.

### Study Objectives

In 7 of the 11 studies [9,18,24,29-32] analyzed, a mobile app was designed and evaluated, whereas 4 studies [23,33-35] evaluated a previously designed app. Furthermore, 7 studies [9,23,29-31,33,35] evaluated both the perceived usefulness and treatment adherence when using a mobile app. In addition, 4 studies assessed perceived usefulness but did not evaluate adherence [18,24,32,35].

Moreover, 1 study [34], in addition to evaluating the mobile app’s viability and acceptance, compared the intervention of 4 groups: mobile app with a reminder, mobile app without a reminder, electronic pillbox with a reminder, and electronic pillbox without a reminder. The objective of another study [18] was to evaluate the ad hoc–designed mobile app and compare the responses between people both older and younger than 55 years. Yet another study [35] compared the ease of use and usefulness of various mobile apps found for managing medication.

### Description of the Population in the Reviewed Studies Using These Mobile Apps

The sample sizes varied between 16 and 99 participants [9,24]. The participants’ ages varied depending upon the study. Of the studies, 2 focused on adolescents [24,29], 1 was directed at persons over the age of 65 years [9], another 1 was for persons over the age of 60 years [31], and another 1 for persons over the age of 50 years [35]. In addition, one study included adults with a wide age range (from 45-90 years) [34] and another compared the responses between people older and younger than 50 years [18].

The investigators recruited the samples at hospitals [23,24,29,30,33], health centers [9,32], patient associations [23,32,34], and local cardiac rehabilitation sports groups in a university [31] as well as with flyers and events at social centers and medical clinics [35].

### Chronic Conditions of the Patients Included in the Studies of Mobile App Use

The apps used in this study included different conditions, such as asthma [29], heart failure [31,34], hypertension [30,33], and HIV [23,33]. The remaining apps did not focus on a specific illness [9,18,24,32,35]; however, the inclusion criteria for 2 of these studies included people suffering from multiple pathologies [9,32,33], and in another of the analyzed studies, the patients had to be recipients of solid organ transplants [24].

### Designs Employed in the Studies

To compare results, 4 studies carried out randomized controlled trials [9,23,31,34]. The first of these compared treatment adherence in 2 groups: those who used the mobile app (experimental group) and those who did not (control group) [9]. The second study that conducted a randomized controlled trial compared treatment adherence between a control group that employed a mobile app with an experimental group that used an extended version of that same app [23]. The third randomized controlled study compared the interventions of 2 groups, mobile app and electronic pillbox, and under 2 conditions each: mobile app with a reminder, mobile app without a reminder, electronic pillbox with a reminder, and electronic pillbox without a reminder [34]. Finally, the fourth study compared the use of the app versus a paper diary [31].

Furthermore, 2 studies compared 2 independent samples. One of these compared the responses of persons older and younger than 55 years as its objective was to verify the differences between the effectiveness and ease of use of the app between these 2 groups [18]. Another compared the effectiveness of various mobile apps [35].

The remaining 5 studies [24,29,30,32,33] described assessment of the mobile apps by the patients

### Time of Use of the Mobile Apps

The time that the participants used the mobile apps varied between 2 hours and 6 months, depending upon the study [33,35].

A description of these issues is in Table 1.

**Table 1:** Details of the included studies.

Authors and country	Objective	Participants	Chronic condition	Design (Duration)
Anglada-Martínez et al, Spain [33]	Evaluate 1 Web and smartphone-based medication self-management platform, named MedPlan.	N=42; average age: 56 years	Hypertension “and” or “or” dyslipidemia and HIV	Transversal (6 months)
Burbank et al, United States [29]	Examine the viability of a mobile application for adolescents with asthma.	N=20; adolescents; average age: 13.5 years	Asthma	Transversal (8 weeks)
Fallah and Yasini, France [18]	Design and evaluate a mobile medication reminder app.	N=60; <55 years: N=30; and >55 years: N=30	—^a^	Transversal (—)
Goldstein et al, United States [34]	Compare the adherence of 2 interventions, electronic pillbox and mobile apps, under experimental conditions with and without medication reminders, in addition to evaluating the viability and effectiveness of each.	N=58; elderly adults; average age: 69 years	Heart failure	Randomized controlled (28 days)
Grindrod et al, Canada [35]	Explore the ease of use and usefulness of existing mobile apps for handing medication in elderly adults.	N=35; >50 years; average age: 67 years	—	Transversal (2 hours)
Kang and Park, South Korea [30]	Develop a mobile application for managing hypertension and evaluate its usefulness, user satisfaction and adherence to medication.	N=38; average age: 56 years	Hypertension	Transversal (4 weeks)
Mertens et al, Germany [31]	Analyze if mobile application to support the therapy management will be accepted by elderly patients with chronic conditions and would improve their therapy adherence.	N=24; average age: 73.8 years	Coronary heart disease or myocardial infarction	Randomized controlled (84 days)
Mira et al, Spain [32]	Design, develop, and evaluate a mobile app that enables safer use of medication in elderly patients who take multiple medications.	N=61; elderly adults; average age: 68.8 years	Pluripathology	Transversal (—)
Mira et al, Spain [9]	Design, implement and evaluate a mobile app for self-management of medication in elderly patients who take multiple medications.	N=99; >65 years; experimental group: N=51; and control group: N=48	Pluripathology	Randomized controlled (3 months)
Perera et al, New Zealand [23]	Examine the effectiveness of a mobile application for facilitating treatment adherence to combined antiretroviral therapy.	N=28; average age: 46; experimental group: N=17; and control group: N=11	HIV	Randomized controlled (3 months)
Shellmer et al, United States [24]	Design a mobile application for improving treatment adherence in adolescent recipients of solid organ transplants and evaluate its acceptance, ease of use and satisfaction.	N=7; adolescents; + 9 caregivers	Recipients of solid organ transplants	Transversal (6 weeks)

^a^Missing data.

### Functions of the Mobile Apps

The contents of the mobile apps included reminders for taking medication; some of these studies did so with alarms (visual and audio) that the patients had previously recorded [9,18,24,29-31,33]. When the alarm sounded, they had to confirm that they had taken the medication [9,23,24,29,31,33], and the apps notified their caregivers when the users failed to indicate that they had indeed taken the medication [9,24]. The apps provided instructions on how to take the medication [9,18,24,31-33], general information about the treatments and medication [18,30-33], education about the illness [24,29], and recommendations on healthy habits [9,30].

Some more specific functions of each app included reminders with alarms for doctors’ appointments [30], blood pressure records [30], or their symptoms in general [29] and images of the medications taken to distinguish them when the time came for them to be taken, thus increasing patient safety [9,31,33]. The TUMEDICINA app (APPANDABOUT, SL) enabled scanning of the bar codes on medication containers to gain information about the intended therapeutic objectives, verbal instructions on how and when to take them, interactions with other medications, expiration dates, and storage indications. All this information was stored as audio recordings [32]. The app for HIV patients contained a 24-hour medication clock for the control and experimental groups. The latter used an extended version of the app that additionally included personalized images about the level of medication and the level of immunoprotection within the patient’s body [23]. The Teen Pocket PATH app had one version for caregivers and another for patients and included general information such as telephone help numbers [24].

### Profile of Mobile Apps Design Participants

In 6 of the studies, the design of the app was made from patient data compiled with qualitative techniques, such as in nominal groups [9,18,24,29,30,32]. In addition, health professionals participated in the app design in 3 of them [9,18,29], and technology specialists also participated in 1 [19]. In another study, in addition to including participation from the patients who were subsequently going to use the app, the design also kept their caregivers in mind [24]. In another study, the app was designed exclusively by technology experts [30].

### Mobile App Availability

In 5 of the 11 studies, the mobile apps were available in both Android and iOS versions [9,29,32,34,35], whereas 4 were only available for Android [18,23,24,30,33] and 2 were only available for iOS [31]. Furthermore, among the studies, 1 study compared mobile apps of Android and iOS environments [35]. In 3 studies, the participants downloaded the app on their mobile phones [23,29,30] and in 4 studies, the users were offered either iPads or tablets with the downloaded app [9,24,31,35].

### Reference Measures for Evaluating Treatment Adherence

The questionnaires administered for evaluating treatment adherence were the Modified Morisky Scale [30]; the Morisky Medication Adherence Scale along with a questionnaire for evaluating the rates of lost doses and medication errors [9]; Medication Adherence Report Scale [23]; Simplified Medication Adherence Questionnaire [33]; the subjective adherence measure A14 scale [31]; the Asthma Control Test for evaluating the impact of asthma on daily functions, frequency of shortness of breath, frequency of asthmatic symptoms at night, frequency of using rescue medicines, and general control of asthma; and the Child Asthma Self-Efficacy for determining the prevention and management of asthma attacks [29]. The other methods used included pharmacy dispensers, measuring the quantity of virus in the blood plasma of each HIV patient [23], and dividing the number of medications that were marked as having been taken by the number of medicines prescribed [34].

#### Quantitative Measures for Evaluating Mobile App Functions

The questions used for evaluating the mobile apps included the Post Study System Usability Questionnaire [24] and the System Usability Scale, which determined the use of the app [35]. One study administered a questionnaire to evaluate the app’s effectiveness and ease of use in which the questions for evaluating the ease were extracted from the System Usability Scale [18]; 1 study created a questionnaire to evaluate the acceptance, usefulness, satisfaction, willingness to recommend the app to other persons, and the opinion the users held about it [34]; and 1 study evaluated the usefulness by using the questionnaire on perceived usefulness by Davis and the satisfaction by means of a questionnaire that evaluated satisfaction with each of the app’s contents [30]. Another study evaluated the use of the app according to the number of times that each participant examined each of the contents in the app and the amount of time invested in each content. Furthermore, a questionnaire was administered with questions about the app’s satisfaction, perceived utility, ease of use, visual appeal, and discretion and about the information it provided [23]. In another study, a questionnaire was administered that evaluated the app’s characteristics and operation [32]. Finally, 1 study assessed usability and satisfaction through self-reported questionnaires [33].

#### Qualitative Measures for Evaluating Mobile App Functions

Overall, 7 studies compiled patient data using qualitative techniques wherein questions were asked about the satisfaction, usefulness, ease of use, acceptance and the contents of the apps [9,24,29,31,32,35].

### Mobile App Effectiveness in Treatment Adherence

Furthermore, 7 studies confirmed that the mobile app increased treatment adherence [9,23,29-31,33,34], and in 5 of them, the differences in adherence before and after the study were statistically significant [9,23,30,31,33]. The study that compared the intervention of the mobile app with that of the electronic pillbox did not find significant differences between the type of device used or the reminders and treatment adherence; however, the participants declared that they preferred the mobile device [34]. Another study did not find statistically significant differences in the control of asthma before and after the study, although the patients with uncontrolled asthma before the study did show a significant increase in their scores. Mean scores on asthma self-efficacy before and after the study increased but were not significant. However, there was a significant increase in preventing an asthma attack [29]. In addition, 3 studies found that the mobile app reduced the occurrence of missed dose significantly [9,31,33]. In addition, the device decreased medication mistakes only in people who had reported committing 2 or more errors before the study [9].

### Satisfaction With the Mobile Apps

The participants declared that that they were satisfied with the app in all 7 of the studies that included this measure [9,23,24,29,30,32,33]. They were more satisfied with the functions that helped them to promote treatment adherence such as reminders and recording symptoms and medication information [30,35]. People who rated the highest were those who organized their medication in pillboxes, took notes on medication containers, and took less than six every day [32]. Moreover, experimental groups who used mobile apps were more satisfied compared with control groups with other devices [23,34].

### Other Evaluated Elements

Ease of use was estimated in 6 studies [9,18,23,24,32,35], and in 4 of them, the participants stated that the app was easy to use [9,18,24,32]. Furthermore, 1 study confirmed that there were no statistically significant differences in the ease of use between those younger and older than 50 years [18], whereas another found no statistically significant differences between persons who used mobile phones or browsed the internet with those who did not [32]. In 1 study that compared various apps, only 1 of the apps received scores for ease of use that were lower than the remaining apps. Moreover, people rated the experience of using the mobile apps as difficult, although that changed when they learned how to use them [35].

In 5 studies, the participants stated that these mobile apps were useful [23,24,30,32,34]. In addition, in 1 study, the participants suggested that the app would be even more useful if it added the option of an alarm as a reminder for taking medication [23]. Yet another study demonstrated that the ideal app would be one that helped foster treatment adherence and, furthermore, provided information about the illness and its treatment [35].

Finally, 1 study [23] that compared a reduced version of an app (control group) with an extended version (experimental group) found that the participants from the experimental group rated their app as more informative, more visually appealing, and more of a motivator for promoting adherence to treatment in comparison with the control group, and almost all the participants would recommend the mobile app to their friends.

A description of these issues is in Table 2.

**Table 2:** Details of the apps used in the included studies.

Study	App functions and design	Medication adherence measure	Measure for evaluating app	App evaluation
Anglada-Martínez et al [33]	MEDPLAN. Drugs information, medication reminder alarm system, where patients confirm whether they have taken the drug or not. App designed by health professionals.	Simplified Medication Adherence Questionnaire (SMAQ), pharmacy refill method and number of days with missing dose.	Usability and satisfaction assessed through self-reported questionnaires.	When adherence was measured using the SMAQ, treatment adherence improved during the intervention phase (19.4%; *P<*.05), and the number of days with missed doses decreased (*P<*.05). The mean satisfaction score for Medplan was 7.2 ± 2.7 out of maximum of 10 points. 71.4% of participants said they would recommend the App to a friend, and 88.1% wanted to continue using it. They thought the application could be more useful in patients on polypharmacy, at the beginning of a treatment, for caregivers or for the elderly population.
Burbank et al [29]	Medication reminder, reminder for recording symptoms, feedback on its adherence and education about asthma. The App was designed by patients and health professionals.	Asthma Control Test. Child Asthma Self-Efficacy Questionnaire.	Questions about satisfaction.	In spite of the improvement in the control of asthma before and after the study, there were no significant differences (*P*=.53). However, the scores improved significantly for those who did not control asthma before the intervention (*P*=.03). Mean scores on self-efficacy before and after the study increased, but were not significant (*P=*.13). Although there were significant differences in preventing an asthma attack (*P=*.04). Satisfaction: 93%
Fallah and Yasini [18]	Reminders via alarms, instructions and information about medication. The App was designed by patients, health professionals, and technology specialists.	—^a^	Questionnaire for evaluating the application’s effectiveness and ease of use. The questions for evaluating its ease of use were taken from the System Usability Scale adapted for mobile applications.	No significant differences were found between the effectiveness or ease of use in either age group (greater and younger than 50). Both groups found the app effective and easy to use.
Goldstein et al [34]	—	Electronic pillbox: opening the pillboxes. Mobile application: electronic self-reports. The number of medications taken was divided by the number of medications prescribed.	Questionnaire for evaluating the acceptance, usefulness, satisfaction, willingness to recommend it and their opinion about the device.	Improves treatment adherence with both interventions (80%). No significant differences were found between the type of device and adherence (*P*=.87), neither were there between the condition and adherence (*P*=.48). Those in the mobile application group awarded higher scores on acceptance and usefulness of their device (*P<*.001). All participants preferred the intervention of the mobile application.
Grindrod, Li and Gates [35]	—	—	System Usability Scale. Questions in a group session: ease of use, user experiences, expected adoption, concerns about the potential for data entry errors, perceived quality of the provided information and preferences for the different characteristics.	The Pocket Pharmacist application received an ease of use score that was significantly lower when compared to the remaining applications (*P*<.001). They initially rated the experience of using the applications as frustrating, although that changed when they learned how to use them. They would use these applications if they someday needed to due to cognitive or health problems. The ideal application would possess characteristics that helped foster adherence and provide information.
Kang and Park [30]	HYPERTENSION MANAGEMENT APP. Reminders with alarms for taking medication and doctor’s appointments, recording blood pressure, recommendations about lifestyle and information on medication. The App was designed by patients and experts.	Modified Morisky Scale.	Questionnaire with a scale from 1 to 5 that evaluated perceived usefulness and satisfaction with each of the application’s contents.	The average scores on adherence increased significantly before and after the study from 4.2 to 5.2 out of a maximum of 6 points (*P*=.001). Perceived usefulness: 3.7. Satisfaction: 3.8 for medication reminders, 3.2 for alarms, 4.3 for recording blood pressure, 3.1 for the information sent, 3.4 for recommendations, and 3.8 for education about medication.
Mertens et al [31]	MEDICATION PLAN. Reminders via alarms, instructions and information about medication. The App was designed by health professionals.	Subjective adherence was determined by the A14-scale. Objective adherence was measured by number of medications each participant had to take each day.	Semistructured interviews.	The mean for subjectively assessed adherence there was a significant increase after the interventional phase from 50 to 54 out of a maximum of 56 points (*P<*.001). The app showed significant adherence for medication intake (*P=*.03). The majority of participants (n=22) stated that they would like to use the medication app in their daily lives.
Mira et al [32]	TUMEDICINA. Scans the bar codes on the medication box to provide information about its therapeutic objective, indications for taking it, interactions with other medications and its date of expiration. This information is stored as audio recordings. The App was designed by patients.	—	Group session and individual questionnaire for evaluating the characteristics and operation of the application.	The characteristics rated highest were the simplicity and clarity of the verbal messages (96.7%), the usefulness of the verbal messages (93.4%) and the clarity of the information provided (95.1%). No significant differences were found in the assessment of the satisfaction between patients with or without experience of using mobile telephones or browsing the Internet (*P=*.88). The people who rated the application the highest were persons who organized their medication in pillboxes, took notes on medication containers and took less than six drugs every day. Satisfaction: 8.3 out of 10.
Mira et al [9]	ALICE. Reminders with alarms for taking medication and carrying out healthy habits, images of drugs, instructions on how to take medication, SMS sent to caregivers in cases where the medication is not taken. The App was designed by patients, health professionals, and technology specialists.	Morisky Medication Adherence Scale. Questionnaire for evaluating rates of missed doses and medication errors.	Questions for evaluating the application: satisfaction, ease of use, performance, usefulness, reliability, acceptance, design, simplicity, accessibility, if they would recommend it and if it afforded them independence.	Treatment adherence improved in the experimental group (28%; *P*<.001) and in a lower rate of omitted doses (27.3%; *P*=.02). The application was not effective in reducing the rate of medication errors, it only decreased in patients who had reported committing 2 or more errors before the study (41.2%). Satisfaction: 8.5 out of 10. Persons without experience of information technologies said that using the application was not complicated.
Perera et al [23]	The application used by the control group contained a 24-hour medication watch. For the experimental group, in addition to the watch, it contained personalized messages about the levels of medication and immunoprotection in the patient’s body.	Medication Adherence Report Scale. Pharmacy prescriptions filled. HIV viral load.	Questionnaire for evaluating the satisfaction, perceived usefulness, ease of use, visual appeal, discretion and provision of information.	Greater treatment adherence in the experimental group according to the scores on the Medication Adherence Report Scale (40%; *P=*.03) and according to the viral load HIV (19%; *P*=.02). However, there were no significant differences in the pharmacy dispensing data (*P=*.18). The experimental group participants were more satisfied with the application than the control group and they rated it as informative, attractive and motivating. 79% said that adding the option of an alarm to remind about taking medication would be useful. 81% of the experimental group would recommend the application.
Shellmer et al [24]	TEEN POCKET PATH. Reminder of what medication must be taken and in what dose, confirmation that it had been taken, information about the type of transplant received and general information, such as telephone help lines. Caregivers received information as to whether the adolescents had taken their medication. The App was designed by patients and caregivers.	—	Post Study System Usability Questionnaire. Questions during one session: ease of use, viability, satisfaction, usefulness, simplicity of the reminder, warning messages sent to the caregivers and perceptions about long-term use of the application.	Users and caregivers found the application easy to use, effective, useful and they were satisfied with it. The caregivers said that they felt less need to constantly ask the adolescents about whether or not they had taken their medication.

^a^Missing data.

## Discussion

### Principal Findings

These results indicate that mobile apps help promote treatment adherence [9,23,29,30,34]. However, when considering the sample size and time of use of the mobile apps under natural conditions, new studies with longer use times than the apps merit consideration to find out whether an accommodation effect exists that has a negative effect upon adherence, for example, after more than 12 or 18 months of using these apps [21,22,25].

One thing to keep in mind is that these studies focused exclusively on the lack of adherence caused involuntarily by the patient. They did not control participant variables of the locus control type or confidence or relationship with health professionals. Users of these apps who voluntarily and consciously rule out following the treatment can use these devices to gain greater credibility with their caregivers or health professionals by indicating in the app that their medication has been taken even when this is not the case. This is the same problem with traditional pillboxes and in research on therapeutic adherence [8].

The gold standard used for determining therapeutic effectives has been based on the use of reports by patients obtained using validated scales and widely used in research on adherence [9,23,29,30]. Only 1 study used a more objective and reliable measure of adherence, that of blood determinations [23].

The majority of patients stated that the mobile apps they had used were easy to use [9,18,24,32] and useful [23,24,30,32,34] and that, additionally, they were satisfied with their ease of use, navigation, and features [9,23,24,29,30,32]. The studies analyzed show that persons aged over 60 years do not encounter difficulties when using these apps and that, therefore, there are no barriers because of age [9,18,32]. In these cases, it should be pointed out that the apps had been designed with the intrinsic characteristics of the target population in mind, such as letter or image sizes [9,18,32]. In addition, it is important to consider that personal characteristics, such as computer literacy, health literacy, mental health status, and cultural background, are related with the use of mHealth apps [36].

It should be noted that in most of the studies, the mobile apps were designed especially for future users [9,18,24,29,30,32]. This indicates that the app design is made according to the needs of patients and has probably contributed not only to their effectiveness but also, above all, to satisfaction with the app.

The main contents in the apps to foster treatment adherence were reminders with alarms for taking the medication [9,18,30], information about the medication [18,30,32], and medication-tracking histories [9,23,25,29].

Although the level of knowledge about the illnesses or their treatments was not controlled in the studies carried out, one could expect that using these apps contributes to greater knowledge about the disease and the drugs that are taken every day. In some cases, these apps include information about drug storage and about potential (the most frequent) drug interactions with other active ingredients or natural products [18,30,32]. This is a relevant aspect because the studies point out that the knowledge patients possess about their medication could be improved, and it should be an objective when these types of apps are designed. Education about treatment is especially important for those who commit more errors in its administration, such as those who use devices such as glucometers or inhalers, and for caregivers of minors [7,8].

Other app functions to promote adhering to the therapeutic regimen were reminders about leading a healthy lifestyle [9,30] or reminders about keeping appointments with physicians [30]. These functions, positively valued by patients, provide added value compared with traditional pillboxes.

This review shows that mobile apps are effective in promoting treatment adherence and that they contribute to patient safety by avoiding errors in the administration of their treatments. Owing to this, health professionals, such as physicians or pharmacists [37], should promote their use by recommending that their patients download them and then monitor how these apps are used, because simply downloading them does not ensure their full use [8,9].

### Limitations

Among the possible limitations of this study, it should be mentioned that despite having carried out the search in the most important databases on medicine, it is probable that other databases were not considered. In addition, although we used a wide range of descriptors to obtain a more precise strategy, there might be a specific keyword from a concrete area that was not controlled.

Furthermore, we did not include articles in languages other than English and Spanish nor did we consider abstracts from conferences.

Another limitation to highlight is the difficulty in compiling the results because of the wide heterogeneity of methodologies and results from the articles that were found.

This study evaluates the effectiveness of mobile apps as a method for overcoming errors by patients in managing medication. However, these mobile apps do not offer alternatives for controlling voluntary nonadherence by patients.

### Comparison With Other Studies

We know that 1 in 5 elderly patients forget to take their medication or make mistakes when doing so [38]. The use of new technologies is a relevant method for overcoming the problems of lack of adherence to treatments, which results in harmful consequences for the health of patients and for those who are elderly. The effectiveness of the mobile apps could be because of the effects that alarms have on forgetfulness, as this is one of the main contributors to the lack of treatment adherence [25], but these apps must also be employed with patient safety in mind, for example, with information on how to avoid drug interactions, with information on how to properly store the medication, or with instructions on which foods the medication can and cannot be taken with.

Most of the studies focused on specific diseases, but all of them had a common approach toward chronic diseases [9,23,24,29,30,32,34]. Previous research found that lack of treatment adherence is more frequent in persons with chronic diseases because of the complexity of therapeutic regimens, regardless of age [5,9,32,36]. For this reason, solutions to lack of treatment adherence caused involuntarily by the patient must be personalized by considering the patient’s profile and the posology, which have a more direct impact on the difficulties of taking medication.

The relevance of using smartphones to foster treatment adherence is also because of their acceptance, ease of use, and affordability [21,26]. These findings justify that elderly people, when the app has been designed with their needs in mind, are not a barrier as some of the reviewed studies suggest.

Park et al [21] found that positive and personalized feedback resulted in positive effects on medication adherence. This is the function that digital pillboxes perform. Personalizing alarms could contribute to their effectiveness and to that effectiveness lasting for longer periods.

Other studies have evaluated the effectiveness of other technological methods by which treatment adherence can be enhanced, such as telephone calls or SMS [20,21,22]. Although these are just as effective, they involve high costs [20,26]. Furthermore, these interventions only take into account reminders for taking the medication, whereas mobile apps provide more content, such as educational interventions [22].

In addition to mobile apps found for fostering treatment adherence, there are also apps for promoting adherence to other therapeutic regimens, with reminders for leading an appropriate lifestyle, reminders for keeping doctors’ appointments, and monitoring other health information (eg, supplements and manage pets), among others [26,38]. The integration of these functions should be considered when designing new apps for virtual pillboxes.

### Future Research

From these results, recommendations for the design of future apps can also be deduced when considering the contents valued highest by the patients. Park et al [37] have found that features appreciated by users are app performance and practical aspects, helpful reminders and notifications, monitoring other health information, versatility of medication information input and display, and supporting health care visits [37]. Standing out among these are the flexible management of alarms that warn about taking medication and education about the type, use of, and precautions about the medication that they take [23,35]. Conversely, these functions have better value for the participants when they use simple interfaces. For this reason, mobile apps are easy to use and people make more use of them [35,39]. Furthermore, it should be emphasized that the future users of the apps must participate in their design to focus on their necessities [9,18,24,29,35,39].

The majority studies included in this review evaluated treatment adherence by validated scales such as the Morisky Medication Adherence Scale [9,30]. Future studies should incorporate objective measures, for example, the most common measure is blood test [23].

In addition, patient safety should be considered in these mobile apps because these help them to manage their medication and they could make mistakes when taking their drugs [9].

Finally, the studies with longer use times of the apps are considered necessary to integrate the mobile apps in their daily routine and examine their effectiveness for treatment adherence in the long term [17-19,22].

### Conclusions

Mobile apps prevent forgetting about medication and incorrect administration and, thus, contribute to patient safety. In the future, these apps should include personalization of the personal conditions and posology of the medication the patient takes.

## Appendix

Multimedia Appendix 1 Levels of evidence and degrees of recommendation.

Multimedia Appendix 2 Assessment of the internal quality of the design of the studies.

## Abbreviations

CRT: controlled and randomized trial
mHealth: mobile health
MEMS: Medication Event Monitoring System
SMS: short message service
