# Supplementary material for: Mobile Apps for Increasing Treatment Adherence: Systematic Review
Source: J Med Internet Res. 2019 Jun 18;21(6):e12505. doi: 10.2196/12505 (PMC6604503; doi:10.2196/12505)
Supplement: Multimedia Appendix 1 [file jmir_v21i6e12505_app1.pdf]

Multimedia Appendix 1. *Levels of evidence and degrees of recommendation.*

**Authors and year**	**Levels of evidence**[a]	**Degrees of recommendation**[b]
Anglada-Martínez et al., 2016 [33]	3	D
Burbank et al., 2015 [29]	3	D
Fallah & Yasini, 2017 [18]	3	D
Goldstein et al., 2014 [34]	2-	-
Grindrod, Li & Gates, 2014 [35]	3	D
Kang & Park, 2016 [30]	3	D
Mertens et al., 2016 [31]	3	D
Mira et al., 2015 [32]	3	D
Mira et al., 2014 [9]	2+	C
Perera, Thomas, Moore, Faasse & Petrie, 2014 [23]	2-	-
Shellmer, Dew, Mazariegos & DeVito, 2016 [24]	3	D

Scottish Intercollegiate Guidelines Network [28]

[a] The levels of evidence were classified as 1++: meta-analyses, systematic reviews of clinical trials or high-quality clinical trials with very little risk of bias; 1+: meta-analyses, systematic reviews of clinical trials or well-conducted clinical trials with little risk of bias; 1-: meta-analyses, systematic reviews of clinical trials or clinical trials with high risk of bias; 2++: systematic reviews of cohort or case-control studies or studies of high-quality diagnostic tests, cohort or case-control studies of high-quality diagnostic tests with very little risk of bias and high probability of establishing a causal relationship; 2+: cohort or case-control studies or studies of well-conducted diagnostic tests with a low risk of bias and a moderate probability of establishing a causal relationship; 2-: cohort or case-control studies with a high risk of bias; 3: non-analytical studies, such as case reports and case series; and 4: expert opinions [28].

[b] The strengths of the recommendations were classified as (A): at least one meta-analysis, systematic review of CRT or a level 1++ CRT, directly applicable to the target population or sufficient evidence deriving from 1+ level studies, directly applicable to the target population and whose results demonstrate overall consistency; (B) sufficient evidence deriving from level 2++ studies, directly applicable to the target population and whose results demonstrate overall consistency. Evidence extrapolated from either 1++ or 1+ level studies; (C) sufficient evidence deriving from level 2+ studies, directly applicable to the target population and whose results demonstrate overall consistency. Evidence extrapolated from level 2++ studies; and (D) evidence from either level 3 or 4. Evidence extrapolated from level 2+ studies [28].
