# Supplementary material for: Mobile Apps for Increasing Treatment Adherence: Systematic Review
Source: J Med Internet Res. 2019 Jun 18;21(6):e12505. doi: 10.2196/12505 (PMC6604503; doi:10.2196/12505)
Supplement: Multimedia Appendix 2 [file jmir_v21i6e12505_app2.pdf]

Multimedia Appendix 2. *Assessment of the internal quality of the design of the studies.*

**Author and year**	**Patient participation in the app design**	**Control of sampling errors**	**Random or convenience sampling**	**Scale of validated measurement**	**The app was used under natural conditions for a period of 3 months or longer**
Anglada-Martínez et al., 2016 [33]	No	Yes	Convenience	Yes	Yes
Burbank et al., 2015 [29]	Yes	No	Convenience	Yes	No
Fallah & Yasini, 2017 [18]	Yes	No	Random	No	-
Goldstein et al., 2014 [34]	They did not design the app	No	Convenience	No	No
Grindrod, Li & Gates, 2014 [35]	They did not design the app	No	Convenience	Yes	No
Kang & Park, 2016 [30]	Yes	Yes	Convenience	Yes	No
Mertens et al., 2016 [31]	No	Yes	Random	Yes	No
Mira et al., 2015 [32]	Yes	No	Convenience	No	-
Mira et al., 2014 [9]	Yes	Yes	Random	No	Yes
Perera, Thomas, Moore, Faasse & Petrie, 2014 [23]	They did not design the app	No	Convenience	No	Yes
Shellmer, Dew, Mazariegos & DeVito, 2016 [24]	Yes	Yes	Convenience	Yes	No
